# Supplementary material for: Cross-Sectional Association of Blood Selenium with Glycemic Biomarkers among U.S. Adults with Normoglycemia in the National Health and Nutrition Examination Survey 2013–2016
Source: Nutrients. 2022 Sep 24;14(19):3972. doi: 10.3390/nu14193972 (PMC9570941; doi:10.3390/nu14193972)
Supplement: Supplementary file 1 [file nutrients-14-03972-s001.zip › nutrients-1909132-supplementary.pdf]

Cross-Sectional Association of Blood Selenium with Glycemic Biomarkers among U.S. Adults with Normoglycemia in the National Health and Nutrition Examination Survey 2013–2016

Supplemental material

Jingli Yang [1,2,†], En Chen [3,†], Cheukling Choi [4], Kayue Chan [4], Qinghua Yang [5], Juwel Rana [6,7], Bo Yang [8], Chuiguo Huang [9], Aimin Yang [9,*] and Kenneth Lo [4,10,*]

[1] College of Earth and Environmental Sciences, Lanzhou University, Lanzhou 730000, China
[2] School of Public Health and Social Work, Queensland University of Technology, Brisbane, QLD 4059, Australia
[3] Department of Clinical Laboratory Medicine, Institution of Microbiology and Infectious Diseases, The First Affiliated Hospital, Hengyang Medical School, University of South China, Hengyang 421001, China
[4] Department of Applied Biology and Chemical Technology, the Hong Kong Polytechnic University, 11 Yuk Choi Road, Hung Hom, Kowloon, Hong Kong SAR, China
[5] Department of Nephrology, Peking University International Hospital, Beijing 102206, China
[6] Department of Epidemiology, Biostatistics and Occupational Health, Faculty of Medicine and Health Sciences, McGill University, Montreal, QC H3A 1G1, Canada
[7] Department of Public Health, School of Health and Life Sciences, North South University, Dhaka 1229, Bangladesh
[8] Department of Epidemiology and Center for Global Cardiometabolic Health, School of Public Health, Brown University, Providence, Rhode Island, 02912, USA
[9] Department of Medicine and Therapeutics, Prince of Wales Hospital, the Chinese University of Hong Kong, Hong Kong SAR, 999077, China
[10] Research Institute for Smart Ageing, The Hong Kong Polytechnic University, 11 Yuk Choi Road, Hung Hom, Kowloon, Hong Kong SAR, China

***** Correspondence: aiminyang@cuhk.edu.hk (Yang, A.); khklo@polyu.edu.hk (Lo, K.)

† These authors contributed equally to this work.

Table S1. Collinearity analysis (variance inflation factor, VIFs) 3

Table S2. Regression coefficients (β) for the association between blood selenium and glycaemic biomarkers stratified by sex 4

Table S3. Regression coefficients (β) for the association between blood selenium and glycaemic biomarkers stratified by hypertension history 5

Table S4. Regression coefficients (β) for the association between blood selenium and glycaemic biomarkers stratified by BMI category 6

Table S5. Regression coefficients (β) for the association between blood selenium and glycaemic biomarkers stratified by age 7

Table S6. Regression coefficients (β) for the association between blood selenium and glycaemic biomarkers stratified by smoking status 8

Table S7. Sensitivity analysis on the association between blood selenium and glycaemic biomarkers by including people with diabetes (total n= 3,226) 9

Table S8. The values of the lowest (Q1) to the highest (Q4) quartiles for blood Se 10

Table S1. Collinearity analysis (variance inflation factor, VIFs) and the lower limit of detections (LLOD)

	VIFs				LLODs
	FPG (mmol/L)	OGTT (mmol/L)	HbA1c (%)	Insulin (uU/ml)	
Cadmium (Cd)	1.06	1.05	1.04	1.06	0.1 μg/L
Manganese (Mn)	1.01	1.01	1.01	1.01	0.99 μg/L
Mercury (Hg)	1.04	1.05	1.03	1.04	0.28 μg/L
Selenium (Se)	1.02	1.03	1.02	1.02	24.48 μg/L
Lead (Pb)	1.08	1.06	1.05	1.08	0.07 ug/dL

Table S2. Regression coefficients (β) for the association between blood selenium and glycaemic biomarkers stratified by sex

	FPG (mmol/L)	OGTT (mmol/L)	HbA1c (%)	Insulin (uU/ml)
Men				
Blood Se (μg/L)				
Q1	0.00 (Reference)	0.00 (Reference)	0.00 (Reference)	0.00 (Reference)
Q2	0.20 (0.09, 0.32)	-0.09 (-0.51, 0.33)	0.04 (-0.02, 0.09)	2.14 (-0.16, 4.44)
Q3	0.23 (0.12, 0.35)	0.08 (-0.33, 0.49)	0.05 (0.00, 0.10)	3.59 (1.33, 5.86)
Q4	0.15 (0.04, 0.26)	0.25 (-0.17, 0.66)	0.03 (-0.02, 0.08)	2.56 (0.30, 4.83)
P for trend	0.02	0.16	0.23	0.02
Log_{10} Se	1.08 (0.31, 1.85)	1.49 (-1.31, 4.30)	0.20 (-0.15, 0.55)	16.33 (1.07, 31.60)
Women				
Blood Se (μg/L)				
Q1	0.00 (Reference)	0.00 (Reference)	0.00 (Reference)	0.00 (Reference)
Q2	0.01 (-0.10, 0.12)	0.45 (0.11, 0.80)	0.00 (-0.05, 0.05)	0.61 (-1.17, 2.39)
Q3	0.01 (-0.11, 0.13)	0.46 (0.07, 0.84)	-0.01 (-0.06, 0.05)	1.19 (-0.73, 3.12)
Q4	0.10 (-0.01, 0.22)	0.36 (-0.01, 0.73)	0.04 (-0.01, 0.09)	2.43 (0.53, 4.33)
P for trend	0.11	0.06	0.20	0.01
Log_{10} Se	0.26 (-0.54, 1.05)	2.04 (-0.51, 4.58)	0.29 (-0.07, 0.64)	12.53 (-0.61, 25.67)
P interaction	0.78	0.57	0.94	0.88

Model was adjusted for age, race, education level, ratio of family income to poverty, smoking status, alcohol drinking status, body mass index, dietary energy, hypertension history, the levels of blood lead, cadmium, manganese and mercury. Q1, <180; Q2, 180 – 194; Q3, 194 – 208; Q4, ⩾208.

Table S3. Regression coefficients (β) for the association between blood selenium and glycaemic biomarkers stratified by hypertension history

	FPG (mmol/L)	OGTT (mmol/L)	HbA1c (%)	Insulin (uU/ml)
Without hypertension				
Blood Se (μg/L)				
Q1	0.00 (Reference)	0.00 (Reference)	0.00 (Reference)	0.00 (Reference)
Q2	0.05 (-0.04, 0.14)	0.20 (-0.12, 0.52)	0.01 (-0.03, 0.05)	1.41 (-0.31, 3.14)
Q3	0.16 (0.07, 0.26)	0.21 (-0.13, 0.55)	0.02 (-0.03, 0.06)	3.39 (1.57, 5.22)
Q4	0.14 (0.05, 0.23)	0.23 (-0.10, 0.56)	0.01 (-0.03, 0.06)	3.15 (1.38, 4.92)
P for trend	<0.01	0.18	0.46	<0.01
Log_{10} Se	0.99 (0.34, 1.64)	1.60 (-0.78, 3.97)	0.14 (-0.14, 0.43)	21.70 (9.19, 34.21)
With hypertension				
Blood Se (μg/L)				
Q1	0.00 (Reference)	0.00 (Reference)	0.00 (Reference)	0.00 (Reference)
Q2	0.17 (0.01, 0.33)	0.12 (-0.4, 0.63)	0.04 (-0.03, 0.11)	1.17 (-1.38, 3.72)
Q3	0.05 (-0.11, 0.21)	0.14 (-0.38, 0.66)	0.04 (-0.03, 0.11)	1.15 (-1.38, 3.69)
Q4	0.07 (-0.1, 0.23)	0.36 (-0.17, 0.89)	0.07 (0.00, 0.14)	0.68 (-1.95, 3.31)
P for trend	0.73	0.19	0.05	0.66
Log_{10} Se	0.22 (-0.83, 1.27)	1.14 (-2.13, 4.41)	0.43 (-0.04, 0.90)	3.63 (-13.22, 20.48)
P interaction	0.09	0.66	0.06	0.11

Model was adjusted for sex, age, race, education level, ratio of family income to poverty, smoking status, alcohol drinking status, body mass index, dietary energy, and the levels of blood lead, cadmium, manganese and mercury. Q1, <180; Q2, 180 – 194; Q3, 194 – 208; Q4, ⩾208.

Table S4. Regression coefficients (β) for the association between blood selenium and glycaemic biomarkers stratified by BMI category

	FPG (mmol/L)	OGTT (mmol/L)	HbA1c (%)	Insulin (uU/ml)
≤25 kg/m^2				
Blood Se (μg/L)				
Q1	0.00 (Reference)	0.00 (Reference)	0.00 (Reference)	0.00 (Reference)
Q2	0.09 (-0.04, 0.23)	0.29 (-0.18, 0.76)	0.04 (-0.01, 0.10)	0.01 (-1.65, 1.66)
Q3	0.09 (-0.05, 0.23)	-0.02 (-0.53, 0.48)	0.01 (-0.05, 0.07)	0.61 (-1.15, 2.38)
Q4	0.16 (0.03, 0.29)	0.27 (-0.20, 0.75)	0.04 (-0.02, 0.10)	1.14 (-0.51, 2.78)
P for trend	0.02	0.41	0.33	0.14
Log_{10} Se	0.51 (-0.33, 1.35)	0.96 (-2.00, 3.92)	0.16 (-0.23, 0.54)	5.75 (-4.88, 16.39)
25.1-29.9 kg/m^2				
Blood Se (μg/L)				
Q1	0.00 (Reference)	0.00 (Reference)	0.00 (Reference)	0.00 (Reference)
Q2	0.12 (-0.03, 0.27)	0.19 (-0.33, 0.72)	0.02 (-0.04, 0.08)	1.83 (-0.49, 4.14)
Q3	0.11 (-0.05, 0.26)	0.37 (-0.17, 0.90)	0.01 (-0.06, 0.07)	2.98 (0.63, 5.32)
Q4	0.07 (-0.08, 0.22)	0.63 (0.10, 1.15)	0.01 (-0.05, 0.08)	1.75 (-0.60, 4.10)
P for trend	0.47	0.01	0.86	0.12
Log_{10} Se	0.83 (-0.20, 1.85)	4.14 (0.50, 7.77)	0.30 (-0.14, 0.73)	16.70 (1.06, 32.35)
≥ 30 kg/m^2				
Blood Se (μg/L)				
Q1	0.00 (Reference)	0.00 (Reference)	0.00 (Reference)	0.00 (Reference)
Q2	0.06 (-0.07, 0.19)	0.29 (-0.14, 0.71)	-0.01 (-0.07, 0.05)	2.96 (-0.14, 6.06)
Q3	0.17 (0.03, 0.31)	0.53 (0.08, 0.97)	0.05 (-0.01, 0.11)	5.25 (2.01, 8.50)
Q4	0.14 (0.00, 0.28)	0.19 (-0.27, 0.65)	0.05 (-0.02, 0.12)	4.99 (1.61, 8.36)
P for trend	0.02	0.25	0.06	<0.01
Log_{10} Se	0.95 (-0.10, 2.00)	1.46 (-2.02, 4.94)	0.31 (-0.15, 0.77)	31.53 (6.07, 56.98)
P interaction	0.90	0.62	0.51	0.02

Model was adjusted for sex, age, race, education level, ratio of family income to poverty, smoking status, alcohol drinking status, dietary energy, hypertension history, the levels of blood lead, cadmium, manganese and mercury. Q1, <180; Q2, 180 – 194; Q3, 194 – 208; Q4, ≥208.

Table S5. Regression coefficients (β) for the association between blood selenium and glycaemic biomarkers stratified by age

	FPG (mmol/L)	OGTT (mmol/L)	HbA1c (%)	Insulin (uU/ml)
18-39 years				
Blood Se (μg/L)				
Q1	0.00 (Reference)	0.00 (Reference)	0.00 (Reference)	0.00 (Reference)
Q2	0.09 (-0.03, 0.21)	0.54 (0.12, 0.96)	0.01 (-0.04, 0.07)	1.74 (-0.81, 4.28)
Q3	0.20 (0.07, 0.32)	0.58 (0.14, 1.02)	0.03 (-0.03, 0.08)	3.64 (0.98, 6.31)
Q4	0.18 (0.06, 0.30)	0.67 (0.24, 1.09)	0.01 (-0.04, 0.07)	2.57 (0.00, 5.14)
P for trend	<0.01	<0.01	0.57	0.03
Log_{10} Se	1.58 (0.68, 2.48)	4.61 (1.40, 7.82)	0.06 (-0.34, 0.46)	19.60 (0.50, 38.71)
40-59 years				
Blood Se (μg/L)				
Q1	0.00 (Reference)	0.00 (Reference)	0.00 (Reference)	0.00 (Reference)
Q2	0.21 (0.07, 0.34)	0.02 (-0.42, 0.46)	0.06 (0.00, 0.12)	1.39 (-0.66, 3.43)
Q3	0.15 (0.01, 0.30)	-0.13 (-0.56, 0.34)	0.04 (-0.02, 0.10)	2.86 (0.71, 5.02)
Q4	0.16 (0.02, 0.30)	0.13 (-0.32, 0.59)	0.07 (0.01, 0.14)	4.22 (2.12, 6.32)
P for trend	0.05	0.68	0.03	<0.01
Log_{10} Se	0.54 (-0.41, 1.48)	0.48 (-2.63, 3.58)	0.56 (0.16, 0.97)	23.12 (8.80, 37.44)
⩾ 60 years				
Blood Se (μg/L)				
Q1	0.00 (Reference)	0.00 (Reference)	0.00 (Reference)	0.00 (Reference)
Q2	-0.05 (-0.24, 0.13)	-0.43 (-1.11, 0.25)	-0.03 (-0.12, 0.05)	1.74 (-1.35, 4.82)
Q3	-0.06 (-0.25, 0.12)	-0.15 (-0.83, 0.53)	0.00 (-0.08, 0.08)	1.57 (-1.49, 4.63)
Q4	0.00 (-0.20, 0.20)	-0.05 (-0.77, 0.67)	0.04 (-0.04, 0.13)	2.24 (-1.04, 5.53)
P for trend	0.90	0.95	0.25	0.20
Log_{10} Se	-0.03 (-1.20, 1.15)	-0.17 (-4.36, 4.02)	0.16 (-0.38, 0.70)	13.01 (-6.53, 32.55)
P interaction	0.07	0.04	0.30	0.84

Model was adjusted for sex, race, education level, ratio of family income to poverty, smoking status, alcohol drinking status, body mass index, dietary energy, hypertension history, the levels of blood lead, cadmium, manganese and mercury. Q1, <180; Q2, 180 – 194; Q3, 194 – 208; Q4, ⩾208.

Table S6. Regression coefficients (β) for the association between blood selenium and glycaemic biomarkers stratified by smoking status

	FPG (mmol/L)	OGTT (mmol/L)	HbA1c (%)	Insulin (uU/ml)
Never smoker				
Blood Se (μg/L)				
Q1	0.00 (Reference)	0.00 (Reference)	0.00 (Reference)	0.00 (Reference)
Q2	0.08 (-0.03, 0.19)	0.41 (0.05, 0.76)	0.01 (-0.04, 0.06)	1.91 (-0.17, 3.98)
Q3	0.10 (0.00, 0.21)	0.27 (-0.10, 0.63)	0.01 (-0.04, 0.06)	2.22 (0.13, 4.31)
Q4	0.07 (-0.05, 0.18)	0.35 (-0.02, 0.72)	0.06 (0.01, 0.11)	2.49 (0.33, 4.65)
P for trend	0.23	0.12	0.02	0.03
Log_{10} Se	0.01 (-0.72, 0.75)	1.39 (-1.08, 3.85)	0.36 (0.02, 0.69)	13.03 (-1.21, 27.27)
Former smoker				
Blood Se (μg/L)				
Q1	0.00 (Reference)	0.00 (Reference)	0.00 (Reference)	0.00 (Reference)
Q2	-0.09 (-0.25, 0.08)	-0.18 (-0.82, 0.46)	0.01 (-0.07, 0.09)	1.81 (-1.44, 5.06)
Q3	0.02 (-0.15, 0.20)	0.26 (-0.40, 0.91)	0.00 (-0.08, 0.08)	3.98 (0.62, 7.34)
Q4	0.02 (-0.15, 0.19)	-0.12 (-0.76, 0.51)	-0.02 (-0.1, 0.06)	3.59 (0.33, 6.85)
P for trend	0.58	0.92	0.50	0.02
Log_{10} Se	0.73 (-0.47, 1.92)	0.35 (-4.20, 4.90)	-0.14 (-0.67, 0.39)	20.59 (-2.72, 43.90)
Current smoker				
Blood Se (μg/L)				
Q1	0.00 (Reference)	0.00 (Reference)	0.00 (Reference)	0.00 (Reference)
Q2	0.23 (0.07, 0.39)	-0.12 (-0.68, 0.45)	0.04 (-0.03, 0.12)	0.47 (-1.95, 2.90)
Q3	0.23 (0.05, 0.40)	-0.06 (-0.69, 0.57)	0.11 (0.03, 0.19)	3.22 (0.51, 5.94)
Q4	0.35 (0.18, 0.52)	0.50 (-0.10, 1.10)	0.03 (-0.05, 0.10)	0.56 (-1.99, 3.11)
P for trend	0.00	0.13	0.28	0.35
Log_{10} Se	2.69 (1.49, 3.89)	2.43 (-1.80, 6.66)	0.39 (-0.14, 0.92)	11.57 (-6.57, 29.70)
P interaction	0.01	0.46	0.76	0.82

Model was adjusted for sex, age, race, education level, ratio of family income to poverty, alcohol drinking status, body mass index, dietary energy, hypertension history, the levels of blood lead, cadmium, manganese and mercury. Q1, <180; Q2, 180 – 194; Q3, 194 – 208; Q4, ⩾208.

Table S7. Sensitivity analysis on the association between blood selenium and glycaemic biomarkers by including people with and without type 2 diabetes (total n= 3,226)

	FPG (mmol/L)	OGTT (mmol/L)	HbA1c (%)	Insulin (uU/ml)
Blood Se (μg/L)				
Q1	0.00 (Reference)	0.00 (Reference)	0.00 (Reference)	0.00 (Reference)
Q2	0.03 (-0.24, 0.30)	0.22 (-0.20, 0.64)	-0.01 (-0.10, 0.09)	0.90 (-1.12, 2.92)
Q3	0.09 (-0.19, 0.37)	0.36 (-0.08, 0.79)	0.07 (-0.02, 0.16)	1.93 (-0.12, 3.98)
Q4	0.29 (0.02, 0.57)	0.61 (0.18, 1.03)	0.08 (-0.01, 0.17)	1.88 (-0.13, 3.90)
P for trend	0.03	<0.01	0.04	0.05
Log_{10} Se	2.20 (0.33, 4.06)	4.05 (1.12, 6.97)	0.91 (0.27, 1.54)	12.14 (-1.66, 25.95)

Model was adjusted for sex, age, race, education level, ratio of family income to poverty, smoking status, alcohol drinking status, body mass index, dietary energy, hypertension history, the levels of blood lead, cadmium, manganese and mercury. Q1, <180; Q2, 180 – 194; Q3, 194 – 209; Q4, ⩾209.

Table S8. The values of the lowest (Q1) to the highest (Q4) quartiles for blood Se

Blood selenium (μg/L)	Q1	Q2	Q4	Q4	Overall
**Overall**					
Mean (SD)	169 (10.5)	188 (3.88)	202 (3.95)	227 (20.7)	196 (24.1)
Median [Min, Max]	171 [105, 181]	188 [181, 195]	201 [195, 209]	221 [209, 388]	195 [105, 388]
**Men**					
Mean (SD)	168 (10.8)	188 (3.74)	202 (3.91)	226 (17.8)	198 (23.3)
Median [Min, Max]	171 [110, 181]	188 [181, 195]	201 [195, 209]	221 [209, 356]	197 [110, 356]
**Women**					
Mean (SD)	169 (10.3)	188 (4.02)	202 (4.01)	228 (24.2)	194 (24.8)
Median [Min, Max]	171 [105, 181]	188 [181, 195]	201 [195, 209]	221 [209, 388]	192 [105, 388]
**18-39 yrs**					
Mean (SD)	169 (9.19)	188 (3.76)	202 (4.12)	225 (14.6)	196 (21.7)
Median [Min, Max]	171 [129, 181]	188 [181, 195]	201 [195, 209]	221 [209, 318]	195 [129, 318]
**40-59 yrs**					
Mean (SD)	169 (11.3)	188 (3.98)	202 (3.80)	229 (24.7)	196 (26.4)
Median [Min, Max]	171 [105, 181]	188 [181, 195]	202 [195, 209]	222 [209, 388]	194 [105, 388]
**>= 60 yrs**					
Mean (SD)	168 (11.3)	188 (3.98)	202 (3.84)	228 (24.1)	195 (25.1)
Median [Min, Max]	171 [120, 181]	189 [181, 195]	201 [196, 209]	220 [209, 356]	195 [120, 356]
**<=25, kg/m^2**					
Mean (SD)	168 (11.3)	188 (3.88)	202 (3.88)	228 (24.8)	195 (26.0)
Median [Min, Max]	171 [110, 181]	188 [181, 195]	201 [195, 209]	221 [209, 377]	194 [110, 377]
**25.1-29.9, kg/m^2**					
Mean (SD)	169 (10.5)	188 (3.87)	202 (3.97)	227 (20.6)	198 (23.7)
Median [Min, Max]	172 [105, 181]	189 [181, 195]	201 [195, 209]	220 [209, 388]	197 [105, 388]
**>=30, kg/m^2**					
Mean (SD)	170 (9.67)	188 (3.90)	202 (4.02)	226 (16.3)	195 (22.6)
Median [Min, Max]	172 [131, 181]	188 [181, 195]	202 [195, 209]	221 [209, 318]	194 [131, 318]
**HYP**					
Mean (SD)	169 (11.1)	188 (4.03)	201 (3.94)	228 (22.0)	197 (25.1)
Median [Min, Max]	172 [110, 181]	188 [181, 195]	201 [195, 209]	222 [209, 377]	196 [110, 377]
**Non-HYP**					
Mean (SD)	169 (10.2)	188 (3.81)	202 (3.95)	226 (19.9)	196 (23.5)
Median [Min, Max]	171 [105, 181]	188 [181, 195]	202 [195, 209]	221 [209, 388]	194 [105, 388]
**Never smoker**					
Mean (SD)	168 (10.2)	188 (3.80)	202 (3.99)	228 (22.0)	196 (24.4)
Median [Min, Max]	170 [105, 181]	188 [181, 195]	202 [195, 209]	221 [209, 388]	195 [105, 388]
**Former smoker**					
Mean (SD)	171 (9.25)	189 (3.81)	202 (3.72)	228 (23.5)	198 (23.9)
Median [Min, Max]	173 [129, 181]	189 [182, 195]	201 [195, 209]	221 [209, 356]	197 [129, 356]
**Current smoker**					
Mean (SD)	168 (11.8)	188 (4.09)	201 (4.09)	224 (13.1)	193 (23.3)
Median [Min, Max]	172 [110, 181]	188 [181, 195]	201 [195, 209]	221 [209, 288]	191 [110, 288]
